# Human Intestinal Mononuclear Phagocytes in Health and Inflammatory Bowel Disease

**DOI:** 10.3389/fimmu.2020.00410

**Published:** 2020-03-18

**Authors:** Charles Caër, Mary Jo Wick

**Affiliations:** Department of Microbiology and Immunology, Institute of Biomedicine, University of Gothenburg, Gothenburg, Sweden

**Keywords:** crohn's disease, ulcerative colitis, intestine, macrophages, dendritic cells

## Abstract

Inflammatory bowel disease (IBD), including Crohn's disease and ulcerative colitis, is a complex immune-mediated disease of the gastrointestinal tract that increases morbidity and negatively influences the quality of life. Intestinal mononuclear phagocytes (MNPs) have a crucial role in maintaining epithelial barrier integrity while controlling pathogen invasion by activating an appropriate immune response. However, in genetically predisposed individuals, uncontrolled immune activation to intestinal flora is thought to underlie the chronic mucosal inflammation that can ultimately result in IBD. Thus, MNPs are involved in fine-tuning mucosal immune system responsiveness and have a critical role in maintaining homeostasis or, potentially, the emergence of IBD. MNPs include monocytes, macrophages and dendritic cells, which are functionally diverse but highly complementary. Despite their crucial role in maintaining intestinal homeostasis, specific functions of human MNP subsets are poorly understood, especially during diseases such as IBD. Here we review the current understanding of MNP ontogeny, as well as the recently identified human intestinal MNP subsets, and discuss their role in health and IBD.

## Introduction

Crohn's disease and ulcerative colitis (UC) are chronic inflammatory disorders of the digestive tract that comprise the term inflammatory bowel disease (IBD) (1, 2). These diseases are complex, severe, and chronic public health problems for which the incidence and prevalence are increasing worldwide (1, 2). Prevalence rates are highest in westernized countries, but ethnic and geographical differences are beginning to fade due to globalization (3, 4). The onset and pathophysiology of IBD are not fully understood, but the current concept is that uncontrolled immune reactivity against intestinal microorganisms combined with environmental factors in genetically predisposed individuals underlie pathogenesis (5–10). Infiltration of pro-inflammatory immune cells into the intestinal mucosa is induced; this releases cytokines and chemokines, creating a vicious circle and perpetuating tissue damage (11). Moreover, IBD is characterized by intestinal microbiota dysbiosis, with a reduction in both bacterial quantity and diversity (10, 12, 13). In some patients, mucosal inflammation is linked to these alterations and to bacteria-derived factors (14–16). However, whether changes in intestinal microbiota is a cause or a consequence of IBD is currently not known (8, 17–19). Finally, the disease course is characterized by repeated cycles of remission and relapse, adding further complexity to disease pathogenesis.

Crohn's disease can involve any part of the digestive tract, but predominantly the terminal ileum, while UC involves only the large intestine, mainly the rectum (1, 2). Generally, the onset of IBD is in young individuals, often 20–30 years old, and most IBD patients have a normal life expectancy thanks to existing treatments. However, despite very low mortality from IBD, morbidity remains a significant problem, and conventional medication involves escalating drug regimens with concomitant side effects. Moreover, IBD is not curable and increases the risk for lymphoma, biliary cancer, and colorectal cancer (20, 21). A significant number of IBD patients do not respond to treatments and must instead undergo surgery to relieve symptoms, often multiple times. Surgery is not only a major procedure for patients, but can also result in postoperative complications and infection, and negatively influences the quality of life (1, 2, 22). Regarding immunomodulators, some, albeit relatively few, targets have been identified; however, there is unfortunately a loss of treatment efficacy over time (1, 2, 11). Moreover, immunoregulation is altered during disease course and flare-ups, which affects treatment timing and efficacy (23). Thus, there is a great need to develop new targeted immunotherapies and, importantly, to identify methods to screen patients for likeliness to respond to a given therapy prior to starting treatment (24–26). To achieve these goals, it is important to further our understanding of IBD immunopathogenesis in humans.

Mononuclear phagocytes (MNPs) consist of multiple specialized innate immune cell types, including monocytes, macrophages (Mfs), and dendritic cells (DCs) (27–29). These cells are central to eliminating pathogens by their ability to sense, internalize and digest microbes and present antigens to T cells to drive adaptive immunity. They also secrete chemokines and cytokines, resulting in the migration and activation of immune cells (30–33). Importantly, both DCs and Mfs collaborate to maintain intestinal tolerance against food antigens and commensal bacteria through the induction and maintenance of regulatory T cells (Tregs) (34–40). Thus, MNPs have critical roles throughout the body in maintaining homeostasis and health. However, inappropriate activation of MNPs can induce sustained inflammation and tissue damage resulting in autoimmune and chronic inflammatory diseases such as IBD (41–47).

Therefore, defining how MNPs control immune homeostasis in the healthy human gastrointestinal tract, and their contribution to the aberrant immunoregulation that results in disease, is critical to improving treatments for IBD patients (7, 48–50).

This review focuses on the current understanding of MNP subset ontogeny as well as their role in the human intestine during health and IBD. Deciphering human intestinal MNP subset characteristics and understanding their roles in tipping the balance from intestinal health to IBD will provide insight for the development of new therapies to reset aberrant cellular functions that drive the chronic inflammation of IBD.

## Ontogeny, Location, and Characterization of MNP Populations

### Monocytes and Macrophages

#### Origin

**Figure 1:**
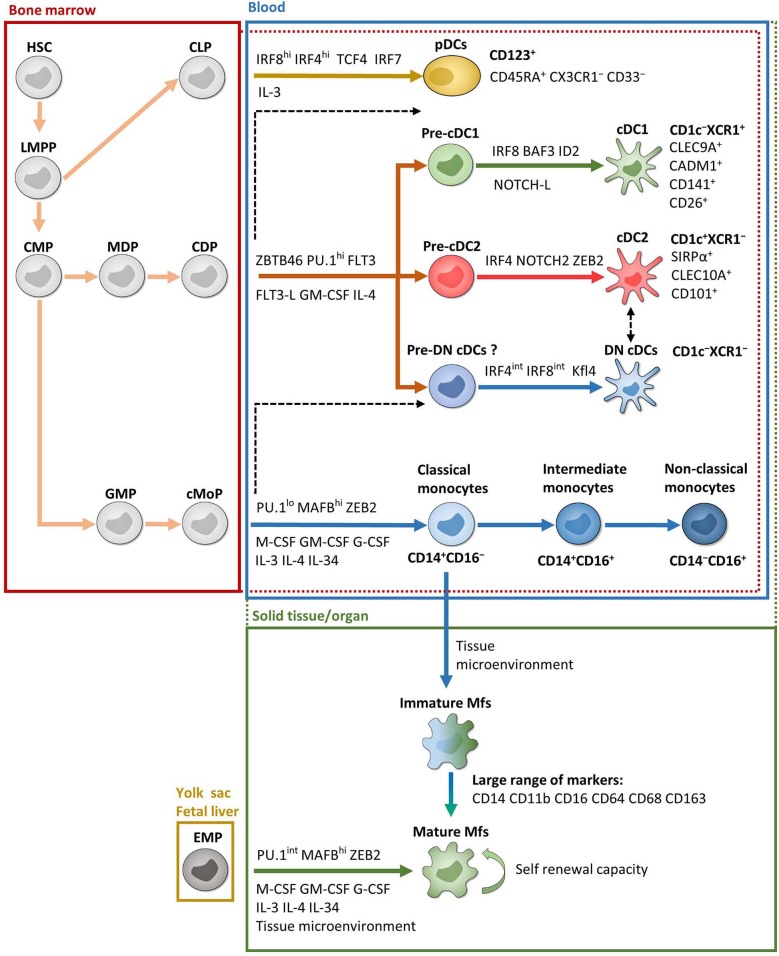
Ontogeny and development of MNPs. Except EMP and pDC ontogenies, which are exclusively from mice, most of the data are from humans. Red and green dotted lines represent possible overlaps between compartments. For example, pre-cDCs are present in both bone marrow and blood and cDCs are present in both blood and solid tissues. Black dashed lines represent possible developmental processes still under debate. CDP, common DC progenitor; CLP, common lymphoid progenitor; cMoP, common monocyte progenitor; CMP, common myeloid progenitor; EMP, erythro-myeloid progenitor; GMP, granulocyte-macrophage progenitor; HSC, hematopoietic stem cell; LMPP, lymphoid-primed multipotent progenitor; MDP, macrophage-DC progenitor. See the main text for other acronyms.

Monocytes are produced in the bone marrow from common monocyte progenitors that derived from common myeloid progenitors (51–54) (Figure 1). They represent 2–8% of leukocytes in the peripheral blood of healthy individuals and constitute a versatile and dynamic cell population, composed of three major subsets: classical, intermediate and non-classical monocytes (55–57) (Figure 1). Classical monocytes circulate for only a day in the bloodstream and transmigrate to peripheral organs where a majority of them differentiate into tissue-specific resident Mfs and DCs following exposure to growth factors, cytokines, and microbial products in the local microenvironment (57, 58). However, a decade of data from mouse models provides evidence that most tissue-resident Mfs are seeded before birth, derived from erythro-myeloid progenitors in the yolk sac during embryonic development; they acquire tissue-specific characteristics through the microenvironment and possess self-renewal capacity (53, 59–67) (Figure 1). Interestingly, recent articles observed equivalent development and characteristics of tissue-resident Mfs in humans (68–70). There are two exceptions, dermal Mfs (53, 71) and intestinal Mfs, in both mice (53, 72–74) and in humans (75), which are continuously reconstituted by blood classical monocytes. Therefore, each organ has its own unique combination of embryonic and adult-derived Mf populations that change throughout life according to immune responses and tissue repair (53, 69, 75–77).

#### Development

In mouse, development of monocytes and Mfs from progenitors depends on essential transcription factors such as PU.1, MAFB, ZEB2, and macrophage colony-stimulating factor (M-CSF) (65, 78–81) (Figure 1). Several other growth factors and interleukins also play a significant role for their maintenance and homeostasis. This includes granulocyte macrophage colony-stimulating factor (GM-CSF), granulocyte colony-stimulating factor (G-CSF), IL-3, IL-4, and IL-34 (41, 82–85). Importantly, a specific combination of transcription factors is required to maintain the tissue-specific identity of Mfs (81, 86). In humans, even if it is more difficult to study cell development, the same transcription factors and growth factors seem to be involved in monocyte and Mf development such as PU.1, MAFB, and M-CSF (87, 88) (Figure 1).

#### Location and Characterization

As stated above, the blood monocyte population has traditionally been divided into three subsets: CD14^+^CD16^−^ classical monocytes, which represent 85% of the monocyte pool, CD14^+^CD16^+^ intermediate monocytes and CD14^−^CD16^+^ non-classical monocytes (55–57) (Figure 1). Each of these subsets possess specific extravasation and cell fate properties and are implicated in distinct functions and diseases (51, 57, 89, 90). Recently, using single-cell RNA sequencing (scRNA-Seq), Villani et al. have observed four blood monocyte subsets as a result of the high heterogeneity of intermediate monocytes (91). Nevertheless, the frequency of these cells is very low and supplementary studies are needed to fully understand their biological relevance. Moreover, two recent studies showed that one of these four subsets was a contamination by NK cells (92, 93).

Regarding human Mfs, the main phenotypic markers used to characterize them are CD14, CD11b, CD16, CD64, CD68, and CD163 depending on the tissue analyzed (Figure 1). Indeed, Mfs are tissue-specific populations, such as alveolar Mfs in lung or Kupffer cells in liver, which acquire and maintain identities according to their local microenvironment (32, 66, 81, 94–96). Moreover, each organ comprises Mf subsets with distinct phenotypes and functions according to their origin, fate and location. Finally, Mfs are essential to maintain tissue homeostasis, clear apoptotic cells, provide immune system regulation, perform tissue remodeling and repair, as well as defend against pathogens (32, 77, 84, 97–101). Characterization of human intestinal Mf subsets is discussed in detail below.

### Dendritic Cells

#### Origin

The DC population is divided into three major subsets according to their ontogeny and functions: conventional DC1 (cDC1), conventional DC2 (cDC2) and plasmacytoid DCs (pDCs) (55, 102). Typically, DCs derive from bone marrow common DC progenitors, which diverge into pre-cDCs and pDCs (103) (Figure 1). Pre-cDCs undergo maturation in the blood, resulting in cDC1 and cDC2, where they have a short lifespan, and some of which transmigrate to organs (103) (Figure 1). Nonetheless, recent conceptual models in hematopoiesis have shaken up DC ontogeny, as reviewed in (51, 102). Indeed, even if earlier studies have shown that pre-cDCs could be programmed to become cDC1 or cDC2 at several steps of their development (104–107), it is now thought that each bone marrow progenitor follows a predestined pathway according to lineage priming that occurs at early stages in development (51, 108–110). Thus, each phenotypically defined population contains cells primed by related yet distinct developmental pathways that share a common transient phenotype. This has been shown both in mouse models (108), as well as in humans (109, 110). In addition, Rodrigues et al. identified two distinct mature pDC subsets in mouse models (111). One pDC subset is derived from common DC progenitors and the other is derived from common lymphoid progenitors and represents the majority of mature pDCs (111) (Figure 1). However, it is currently unknown whether these two subsets of mature pDCs also exist in humans. Moreover, Dress et al. have recently claimed that all pDCs are derived from common lymphoid progenitors and could be called “plasmacytoid innate lymphoid cells” (112). Thus, although the recent development of scRNA-Seq analysis has led to better understanding of DC subset origins, much work remains, especially in humans.

#### Development

In mouse, DC development is dependent on transcription factors ZBTB46 and PU.1, as well as FLT3 and its ligand, GM-CSF and IL-4 (80, 113–115) (Figure 1). Further development of each DC subset then involves specific transcription factors such as interferon regulatory factor (IRF) 4 and IRF8. More precisely, pDCs require both IRF4 and IRF8 while cDC1 and cDC2 require IRF8 and IRF4, respectively (102, 103, 116–119). Other factors are involved in DC subset development and sustention including BATF3 and ID2 for cDC1, NOTCH2 and ZEB2 for cDC2, and TCF4 (also known as E2-2) and IRF7 for pDCs (43, 45, 111, 120–124) (Figure 1). In humans, the same factors are involved in DC development, particularly PU.1, FLT3 and GM-CSF (87, 106, 107, 125–127), as well as DC subset development via IRF4, IRF8, BATF3, ID2, TCF4, and IRF4 (103, 115). Recently, two studies have shown that adding NOTCH ligands to FLT3L in bone marrow precursor cultures increased the yield of cDC1 and that these NOTCH-cDC1 were transcriptionally and functionally closer to *in vivo* cDC1 (127–129) (Figure 1). However, transcription factor dependency differs considerably between tissues, and the question remains whether this specificity is enforced at the precursor level in the bone marrow or if microenvironmental cues in the organs are the primary regulators of the final steps in DC development (124, 130). This phenomenon seems to be tissue-specific (131). Indeed, Heidkamp et al. showed that DC subsets in lymphohematopoietic organs, i.e., spleen, thymus and blood, are strongly defined by ontogeny rather than by signals from the microenvironment, while it is the opposite in DC subsets from lung or skin (131).

#### Location and Characterization

First, among PBMCs, DCs are identified as CD14^−^CD16^−^ cells among MNPs, i.e., CD45^+^Lin^−(CD3/CD19/CD56)^HLA-DR^+^ cells (132, 133). Then among DCs, cDCs are CD11c^int−hi^ while pDCs are CD11c^−^ (91).

The cDC2 subset is characterized by CD1c and SIRPα among cDCs (91, 118, 131, 134, 135) (Figure 1). CD1c is a glycoprotein involved in the presentation of lipid antigens while SIRPα is an inhibitory receptor, mainly expressed by myeloid cells (136). While all SIRPα^+^ cDCs comprise IRF4^+^IRF8^−^ *bona fide* cDC2 in mouse, two populations of SIRPα^+^ cDCs have been detected in humans: a population of *bona fide* cDC2 with a CD1c^+^IRF4^+^IRF8^−^phenotype and a population of CD1c^−^ cDCs showing the typical IRF4^int^IRF8^int^ expression observed in the monocyte-macrophage population (118). Therefore, CD1c is required to define human *bona fide* cDC2 (Figure 1). In mice, cDC2 are specialized in CD4^+^ naïve T cell polarization in LNs (137, 138). On the contrary, in humans, cDC2 do not have an enhanced capacity to prime CD4^+^ T cells compared to cDC1 (139, 140).

The cDC1 subset was first described as CD141^+^ cells among cDCs (55, 141, 142). However, although CD141 is associated with cDC1, it is also expressed by other blood MNP subsets, including pDCs (91). Moreover, several human tissues contain a CD141^+^CD1c^+^ double-positive population (143, 144), which has been associated with either cDC2 (135) or cDC1 (91). This makes the subset identity of this double-positive population unclear. Fortunately, transcriptional profiling identified new markers that better define cDC1 and can be used for subset confirmation. Such markers include CLEC9A (also called DNGR-1), CADM1, CD26, and CD13 (91, 118, 134, 135, 145–147) (Figure 1). XCR1, a receptor for XCL1 and XCL2 chemokines, can also be used and is conserved in many species (91, 118, 134, 148, 149) (Figure 1). Actually, XCR1^+^ cDCs seem to be the “final form” of cDC1 subset development (127). Indeed, Balan et al. showed that the blood CADM1^+^CD141^+^CLEC9A^+^XCR1^−^ DC fraction proliferates and acquires XCR1 expression during culture, suggesting that these cells are the immediate precursors of the XCR1^+^ cDC1 (127). Moreover, lack of expression of monocyte-macrophage and cDC2 markers such as CD14, CD1c, CD11b and SIRPα is also important to thoroughly identify the cDC1 population. Finally, as some cDC1 have intermediate CD11c expression, caution needs to be used to include all cDC1 by gating cDCs as CD11c^int−hi^ cells (102, 135, 143). Functionally, cDC1 are involved in CD8^+^ T cell priming through antigen cross-presentation as well as in CD4^+^ Th1 and Treg polarization (150, 151). They also seem optimal for the generation of tissue-resident memory T cells, but not for circulating memory T cells, during viral infection, at least in mouse models (152). Thus, the cDC1 population constitutes an interesting DC subset for the design of immunotherapeutic treatments against intracellular pathogens or cancer cells. However, in humans, cross-presentation is also done by cDC2, monocyte-derived cDCs and monocyte-derived Mfs (140, 153–156). Nevertheless, it has been demonstrated that only cDC1 have the capacity to cross-present antigens from necrotic cells (157). Unfortunately, the human cDC1 subset is rare in blood and tissues (135), making it difficult to study them *ex vivo*. Thus, the division of labor between cDC1 and cDC2 subsets is still not fully understood (158), but their physical location in the LNs could contribute to differences in T cell activation, as recently reviewed (159, 160).

There is also a cDC population double negative for CD1c and XCR1 among cDCs (called DN cDCs hereafter), which could be a third *bona fide* cDC subset or a monocyte-derived cell type (Figure 1). This population is also present in several organs (118, 135); however, little is known about their functions or their involvement in diseases. Thus, further investigation is needed to fully characterize this DN cDC population.

Finally, it has recently been shown that the traditional gating strategy characterizing human pDCs, which is CD123^+^ cells among CD14^−^CD11c^−^ MNPs, also includes pre-cDCs (91, 103). Thus, additional markers such as CD45RA, CX3CR1, and CD33 are required to analyze *bona fide* pDCs (103) (Figure 1). Concerning their functionally, pDCs are mostly involved in antiviral responses through the secretion of type I IFNs (102, 122, 161).

### Monocyte-Derived Cells

The fate of monocyte-derived cells is an area of active research and contains issues that are actively debated (51, 130, 162, 163). Indeed, it is now clear that blood DCs and blood monocytes arise from bone marrow precursors (Figure 1). In tissues, Mfs can arise from both embryogenic precursors and blood monocytes while DCs can arise from blood pre-DCs, blood DCs, tissue pre-DCs or even blood monocytes (Figure 1). Thus, the origin of tissue Mfs and DCs are multiple and complex and also depend on the tissue type as well the inflammatory and wound healing status (35, 58, 164, 165). Therefore, phenotype and functions of these cells during tissue homeostasis and their alterations during disease are not fully understood.

In human tissues, Segura and colleagues have suggested that HLA-DR^+^CD14^+^CD1c^+^ monocyte-derived cells, which increase during inflammation, are inflammatory DCs (162, 166, 167). These cells display a typical DC morphology and possess hallmark DC functions, such as the ability to stimulate naïve T cells (162). However, these CD14^+^CD1c^+^ monocyte-derived cells also expressed markers found on Mfs, including CD64 (166, 167). In addition, Schrøder et al. have recently shown that a fraction of CD14^+^ monocytes already expressed CD1c in blood (168). These CD14^+^CD1c^+^ cells characterized by Schrøder et al. possess hallmarks of monocytes such as CCR2 expression, TNF induction after LPS treatment and lower efficiency to promote naïve T cell proliferation compared to blood CD14^−^CD1c^+^ cDCs (168). Together, these data support that tissue CD14^+^CD1c^+^ monocyte-derived cells could represent a highly plastic Mf subset, which shared some capacities with cDCs, rather being than a *bona fide* cDC subset (130, 169). These data underscore that nomenclature within the MNP compartment should be based on ontogeny rather than phenotypic characterization. Thus, labeling a cell as a “DC” should be restricted to cells derived from dedicated precursors, pre-DCs. Consequently, CD14^+^CD1c^+^ cells with DC-like functions should be referred to as monocyte-derived cells rather than CD14^+^ DCs (163).

To note, a new twist has come from a recent publication by Ginhoux's lab, where data indicate that human inflammatory CD14^+^ DC3, a subset of blood cDC2, are not monocyte-derived cells, but are FLT3L responsive and rather belong to DC lineage (92). In addition, the most inflammatory CD14^+^ DC3 subset, namely CD163^+^CD14^+^ DC3, increase in the blood of patients with systemic lupus erythematosus (92). Therefore, the use of powerful single-cell techniques will likely continue to add to the depth and breadth, as well as the complexity, of the seemingly ever-expanding human MNP family.

Nevertheless, monocyte-derived cells, which have high plasticity, and *bona fide* cDCs are synergistic close collaborators that complement each other in time and space and work toward the same goal—the clearance of pathogens without inducing an immunopathological response (130). Furthermore, DC heterogeneity is highly variable among individuals (133), and surface markers, TLR repertoire and functions of MNP subsets are tissue-specific, as discussed above. Therefore, the continuing delineation of MNP subsets underscores the ongoing need to determine the specific functions of these cells to better understand the development and propagation of diseases such as IBD.

## Human Intestinal Homeostasis and Disruption During IBD

### Homeostasis

**Figure 2:**
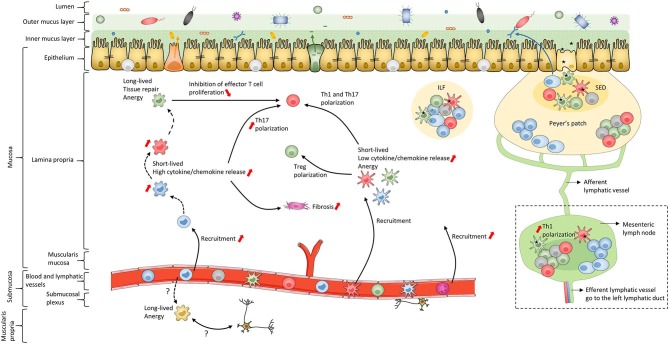
Schematic of human intestinal mucosa and submucosa structure. The schematic is an overall representation of intestinal organization. For example, only the small intestine has Peyer's patches while only the colon has both inner and outer mucus layers composed of firm and loose mucus, respectively. In addition, this schematic does not represent the villi or the crypts of the intestinal mucosa. The color coding of macrophage subsets and dendritic cell subsets matches that in Figures 1, 3, 4 and 6 to allow continuity in the Figures. Black dashed lines represent the maturation waterfall of macrophages. Questions marks represent remaining unresolved issues regarding differentiation and function of the Mf4 subset. Red arrows show processes that increase or decrease during IBD. ILF, isolated lymphoid follicle; SED, subepithelial dome.

In addition to the skin, the intestine is one of the major interfaces with the external environment; it is in contact with pathogens as well as commensal microbiota and food antigens (Figure 2). To maintain homeostasis, this bodily niche thus requires a balance between immune tolerance and immune responses against pathogens (170–182). The intestinal epithelium, mainly composed of a single-cell layer of enterocytes, forms a critical continuous physical barrier with tight junctions connecting adjacent cells and regulates selective permeability for luminal content (Figure 2) (183, 184). In addition to this physical barrier, stem cells located at the base of intestinal crypts (185) continuously give rise to several other epithelial cell types that are involved in specialized functions to maintain homeostasis (186, 187). This includes Paneth cells (188) and goblet cells (189) that secrete antimicrobial peptides and mucins, respectively (Figure 2). The small intestine has a single mucus layer while the colon has an inner mucus layer, lacking bacteria, and an outer mucus layer, which forms a habitat for numerous microorganisms (189) (Figure 2). Despite these systems, luminal antigens can cross the epithelial barrier using one or more routes, such as microfold cells in Peyer's patches, as recently reviewed (50, 190, 191) (Figure 2). Subsequently, antigens come in contact with immune cells, including MNPs, in secondary and tertiary lymphoid organs in the lamina propria (LP) (192, 193). After internalization by MNPs, processed antigens are presented to lymphocytes to induce oral tolerance (173, 191, 194–196) and thus establish homeostatic interaction with dietary factors and intestinal microbiota (37–40, 197). To this end, in addition to their interaction in solitary intestinal lymphoid tissues and Peyer's patches, cDCs are able to migrate to mesenteric lymph nodes (mLNs) through afferent lymphatic vessels to polarize naïve T cells (198) (Figure 2). In contrast, Mfs lack active migratory properties and rather contribute to amplifying T cell responses in the LP. Additionally, intestinal Mfs maintain tissue homeostasis by removing apoptotic and dead cells, remodeling the epithelium and secreting cytokines to sustain Treg functions (32, 35, 38, 42, 45, 71, 98, 199–204). These active regulatory processes, as well as deletion and anergy of T cells, have been implicated in maintaining oral tolerance (191, 205–207). Finally, in response to microbial sensing, cDCs also favor class switching of IgM and IgG to IgA on B cells (208, 209). This is essential for gut homeostasis as IgA transcytoses across the epithelial cell layer to restrain interaction between microorganisms and epithelial cells (210) (Figure 2). To conclude, MNPs control intestinal homeostasis by maintaining immune tolerance to diet- and commensal-antigens while sustaining the capacity to trigger immune responses against pathogens (38, 42, 194, 201, 211). Ideally, these immune responses are a self-limiting process that leads to a complete resolution of inflammation and rapid return to tissue homeostasis. Unfortunately, repeated and aberrant activation of the immune system can result in a chronic inflammatory microenvironment leading to IBD.

### Disruption of Intestinal Homeostasis Through MNPs During IBD

Several defects in intestinal homeostasis have been linked to IBD. These include immune responses against commensal bacteria, epithelial barrier dysfunction, diminution of nutrient absorption, alterations in tissue oxygenation and in autophagy, which induce immune cell recruitment such as MNPs (25, 184, 187, 191, 212–217). In addition, expression of genes associated with variation in Crohn's disease prognosis can be mapped to MNPs (218). Thus, MNPs have a key role in cellular signaling pathways that modulate tolerance vs. chronic inflammation during IBD, as described in detail below (Figure 2).

## Human Intestinal Mucosa MNP Populations During Homeostasis and IBD

First, we summarize MNP subset characteristics and their functions during homeostasis, as assessed in tissue from control individuals, and thereafter we describe MNP alterations during IBD. To note, several layers add complexity to this field, such as various surface markers and gating strategies used in different studies, low number of cells available to perform functional analyzes, and differences between intestinal regions such as the ileum and colon (219, 220). Finally, a caveat to samples used as “controls” is that they are typically from colorectal cancer patients or obese patients.

### Macrophages in Intestinal Mucosa During Homeostasis

#### Phenotype and Frequency of Intestinal Mucosa Macrophages

**Figure 3:**
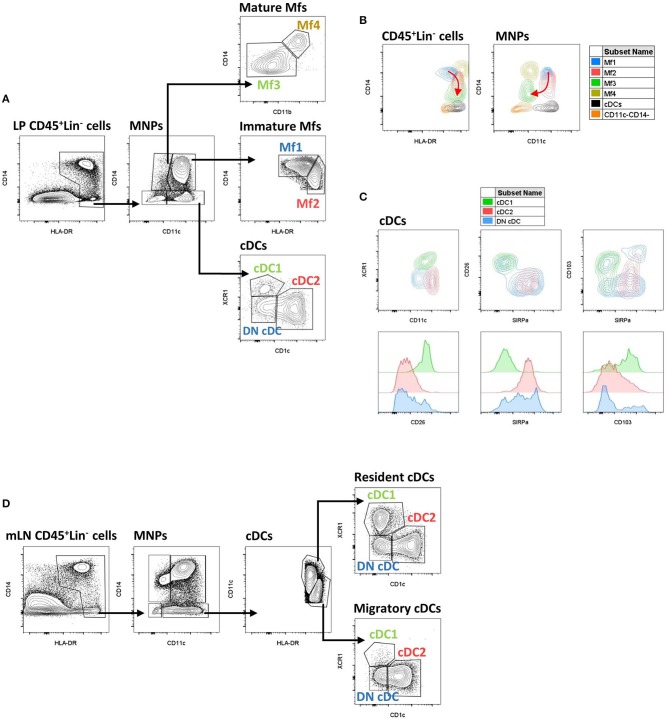
Gating strategy and phenotypic characteristics of human intestinal mucosa MNP subsets and human mLN cDC subsets. **(A)** Gating strategy to analyze human intestinal mucosa MNP subsets from lamina propria cells. This example is from ileum of a Crohn's disease patient. Lineage is composed of CD3, CD19, and CD56. **(B)** Expression level of HLA-DR, CD14, and CD11c on human intestinal MNP subsets. The red arrow indicates the Mf maturation waterfall from Mfl to Mf3. **(A,B)** Based on reference (75). **(C)** Expression level of CD11c, SIRPα, CD26, CD103, and XCR1 on human intestinal cDC subsets. **(D)** Gating strategy to analyze human cDC subsets from mesenteric lymph node cells. This example is from mLN of a ulcerative colitis patient. Lineage is composed of CD3, CD19, and CD56. LP, lamina propria; mLN, mesenteric lymph node.

**Figure 4:**
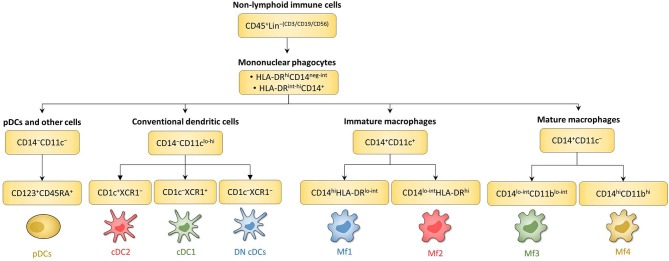
Schematic overview of human intestinal MNP subsets. Main surface markers to identify human intestinal MNP subsets using the gating strategy in Figure 3. This schematic is not intended to show the ontogenic or developmental relationship between the human intestinal MNP subsets.

**Figure 5:**
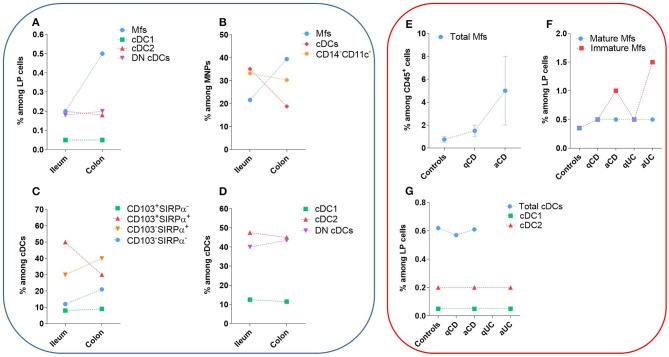
Frequency of human intestinal MNP subsets in homeostasis and during IBD. These values do not take in account the number of patients or the standard error of the mean from each study. The dashed lines are only to clarify the difference between the type of tissue and do not represent paired values. **(A–D)** Frequency of human intestinal MNP subsets in homeostasis. **(E–G)** Comparison of human intestinal MNP subset frequencies in homeostasis and during IBD. a, active lesion areas; q, quiescent lesion areas. References: **(A)** (132, 225); **(B)** (226, 227); **(C)** (75, 134, 220); **(D)** (132); **(E)** (223, 228); **(F)** (132, 225, 228); **(G)** (132, 229).

The first step to characterize intestinal Mfs by flow cytometry is gating on MNPs, i.e., CD45^+^Lin^−(CD3/CD19/CD56)^HLA-DR^int−hi^CD14^neg−hi^ cells (Figures 3A, 4). Then, among MNPs, CD14 (Figures 3A, 4), CD64 and CD163 are used to distinguish Mfs from DCs (72, 75, 221–225). To note, various studies have shown that CD64 alone was not sufficient to distinguish intestinal Mfs from DCs, as some cDC2 are CD64^+^ (72, 135, 220, 224). In control intestinal mucosa, several studies showed that Mfs, i.e., CD14^+^ cells, represent ~0.2 and 0.5% of LP cells (Figure 5A), and 20 and 40% of MNPs (Figure 5B), in ileum and in colon, respectively (132, 225–227). This suggests that the frequency of Mfs is higher in colon than in ileum. However, Granot et al. showed the contrary by analyzing CD14^+^ cells among CD45^+^ cells in organ donors (135), underscoring once again the complexity of working with human tissues.

**Figure 6:**
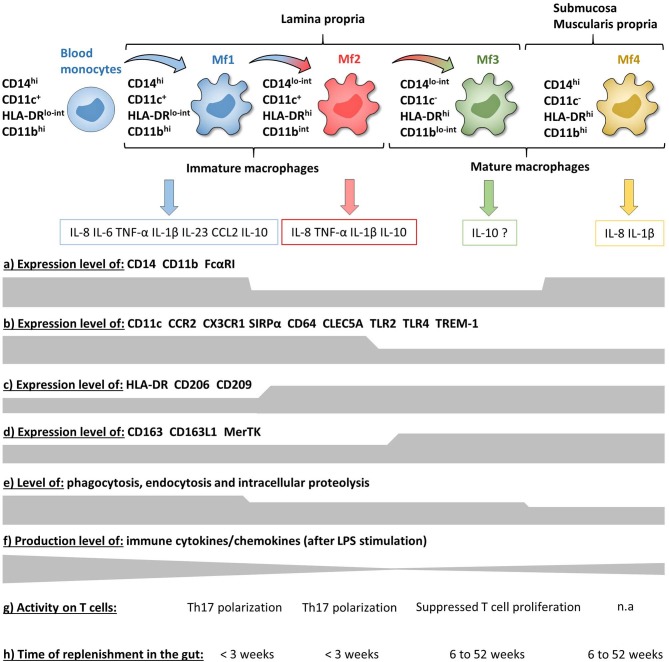
Phenotypic and functional characteristics of human intestinal mucosa Mf subsets during homeostasis. **(a–d)** Expression level of major surface markers on blood monocytes and intestinal mucosa Mf subsets assessed by flow cytometry (intensity level from blood monocytes to Mf4 subset). **(e–g)** Functional characteristics of blood monocytes and intestinal mucosa Mf subsets assessed *ex vivo* (intensity level from blood monocytes to Mf4 subset). **(h)** Time of replenishment of intestinal mucosa Mf subsets assessed in duodenum-pancreas transplanted patients. n.a, not analyzed.

Concerning Mf subsets, it is now well-defined that the human intestinal Mf population comprises a continuum of blood monocyte-derived cells differentiating first into a newly recruited monocyte population (called immature Mfs hereafter) and then into a mature Mf population, similar to the situation in mouse intestine (42, 72, 73, 75, 221). Based on flow cytometry expression of HLA-DR, CD14, CD11c and CD11b, Bujko et al. recently described four Mf subsets (75). More precisely, they showed that both immature and mature Mf populations are composed of two subsets, Mf1 and Mf2, and Mf3 and Mf4, respectively (75) (Figures 3A,B, 4, 6). These four Mf subsets are comparable with those described in human ileum by Bain et al. using HLA-DR, CD14, CD11c, CD163, and CD209 (221) (Figure 6). The Mf maturation waterfall from Mf1 to Mf3 includes a decreased expression of a set of blood monocyte markers such as CD11c and CCR2, as well as an increased expression of CD163 and CD209 (75) (Figures 3B, 6). The Mf4 population is primarily located deep in the mucosa and in the densely innervated submucosa and muscularis propria/externa (75) (Figures 2, 6). Thus, the Mf4 population might be specialized in nervous system interactions, as described in mice (195, 230–233). However, further investigation is needed to better understand the origin and function of these cells in the human intestine.

As previously shown by Jahnsen's group (75, 168, 234), Bernardo et al. confirmed that immature Mfs are characterized as CD11c^+^ Mfs and expressed monocyte-associated markers such as CCR2 and CX3CR1, while mature Mfs are characterized as CD11c^−^ Mfs (225) (Figure 6). However, they did not find a difference of CD206 and CD163 expression between Mf populations (225), which are markers typically associated with mature Mfs (75, 221) (Figure 6). Regarding frequency, mature Mfs were the most abundant Mf population in duodenum but decreased from duodenum to colon while the opposite was observed for the frequency of immature Mfs (75, 225).

To summarize, circulating classical monocytes enter in the intestinal mucosa and differentiate *in situ*, first into transient immature Mfs and then to mature Mfs. The Mf maturation waterfall is based on stepwise acquisition of a set of markers related to bacteria clearance concomitant with loss of inflammatory markers (Figure 6).

#### Function of Intestinal Mucosa Macrophages

Intestinal mucosal Mfs are strategically located in the subepithelial area where they regulate lumen-derived commensal microbe penetrance through their capacities of phagocytosis and degradation. Thus, intestinal mucosal Mfs are involved in pathogen clearance and immune regulation by maintaining tolerance to commensal microbiota and food antigens as well as tissue repair (42, 195, 235–237). The three intestinal mucosal Mf subsets described by Bujko et al. are highly proficient at endocytosis, antigen uptake, and intracellular degradation of proteins, although less than blood monocytes, while the submucosal Mf4 population is weakly competent in these capacities (75) (Figure 6). Compared with the other intestinal Mf subsets, unstimulated and LPS stimulated Mf1 release significantly higher amounts of numerous chemokines and cytokines (75, 225) (Figure 6). To note, the frequency of blood monocyte-derived CD11c^−^ Mfs, related to intestinal mature Mf3, increased in the presence of mucosa-conditioned medium from control intestine (225). In addition, Maheshwari et al. showed that blood monocyte-derived Mfs developed LPS tolerance on exposure to mucosa-conditioned medium, mainly provided by TGF-β2 (238). This suggests that intestinal mature CD11c^−^ Mfs are generated from newly recruited monocytes through the intestinal microenvironment, which promotes an anti-inflammatory and anergic state during the Mf maturation waterfall. This would result in mature Mfs that are less responsive against food antigens and commensal microbiota. Indeed, several studies have shown that, contrary to monocytes and most other tissue-resident Mfs, intestinal Mfs lack surface expression of TLR2, TLR4 and FcαRI, and are consequently down-regulated for LPS- and IgA-mediated activities (75, 239, 240) (Figure 6). Moreover, mature Mfs reduce the release of proinflammatory molecules together with desensitization to TLR ligands, which is a functional feature of anergy (75, 239, 241, 242). This anergic status is thought to be driven by several micro-environmental factors, as recently reviewed (237), such as TGF-β, which induces downregulation of the MyD88 pathway in blood monocytes and results in a hyporesponsive mature Mf-like phenotype (242, 243). Confirming this, Kelly et al. recently showed that human intestinal Mfs highly expressed integrin αvβ8, which down-regulated TNF production via TGF-β activation, at least in blood monocytes (244). Some earlier studies also support the hyporesponsive nature of intestinal Mfs. Indeed, more than 10 years ago, using an unconventional Mf definition, i.e., CD13^+^CD14^−^CD33^−^CD64^−^CD16^−^ cells, Smith et al. showed that even if jejunum Mfs had strong basal phagocytic and bactericidal activities they were anergic (239, 242, 245). More precisely, they did not secrete cytokines and chemokines, except low amounts of IL-8, with or without LPS stimulation (239, 242) and did not activate the NF-kB pathway (243). However, in contrast, several recent studies showed that intestinal Mfs, defined more conventionally as CD14^+^ cells, express TLRs and secrete pro-inflammatory molecules with or without TLR stimulation (75, 226, 227, 246). Indeed, intestinal Mfs from controls produced few cytokines in the absence of stimulation while secretion of IL-12p40, IL-23, TNF, IL-6, and IL-10 were induced by commensal bacteria (246). More specifically, Mfs were the major producers of IFN-γ, TNF, IL-6, TL1A, and IL-23 among LP cells activated or not by commensal bacteria (246, 247). Furthermore, Mf-derived IL-23 induced IFN-γ and TNF release from LP cells (246) as well as IL-22 secretion by innate lymphoid cells (248).

Human intestinal Mfs also express a receptor called TREM-1 (triggering receptor expressed on myeloid cells 1), which amplifies inflammatory responses upon engagement by thus far poorly understood ligands (249–251). TREM-1 activation leads to the secretion of pro-inflammatory cytokines and chemokines such as TNF, IL-6, IL-1β, IL-8 and CCL2, and synergizes with TLR activation (251–254). Our group and another showed that intestinal mature Mfs expressed less TREM-1 than immature Mfs and blood monocytes (253, 255), which has been corroborated by a recent study using scRNA-Seq (228) (Figure 6). The decrease of TREM-1 expression during Mf maturation seems to be due to the intestinal anti-inflammatory microenvironment, as monocytes cultured in the presence of IL-10 and TGF-β have reduced TREM-1 expression (253).

Takeda's group described three human intestinal Mf subsets as CD163^lo^, CD163^hi^CD160^lo^, and CD163^hi^CD160^hi^ (226, 256). They showed that CD163^lo^ Mfs, which seem related to Mf1, highly secreted pro-inflammatory cytokines, while the CD163^hi^CD160^hi^ Mf subset, which seem related to mature Mfs, secreted IL-10 (226, 256). These data corroborate those from Bernardo et al. (225). On the contrary, Bujko et al. showed that, in addition to their high pro-inflammatory cytokine release, immature Mfs, mainly Mf1, secreted more IL-10 than mature Mfs (75). Thus, which subset of intestinal Mfs is the main producer of IL-10 is still an open question (Figure 6). Nevertheless, regarding T cell induction, the CD163^lo^ Mfs (immature Mf1-like) induced naïve CD4^+^ T cell polarization into Th17 cells while CD163^hi^CD160^hi^ Mfs (mature Mf3-like) suppressed effector T cell proliferation (226, 256) (Figures 2, 6). However, none of these Mf subsets induced naïve CD4^+^ T cell polarization into Th1 or Tregs (226, 256). This is consistent with data from Matsuno et al. who showed that Mfs could polarize naïve CD4^+^ T cells into Th17, but not into Th1 or Tregs (227). To note, intestinal Mfs do not express CCR7 (135, 222, 246), suggesting that they cannot migrate to mLNs to interact with naïve T cells *in situ*. Therefore, given that human intestinal T cells are almost entirely memory T cells (257–259), the biological significance of the ability of intestinal Mfs to regulate naïve CD4^+^ T cells as shown *ex vivo* is not clear.

Finally, Bujko et al. have also analyzed the replenishment of the four Mf subsets from pancreatico-duodenal transplantation patients (75). Three weeks after surgery almost all donor Mf1 and Mf2 were replaced by recipient Mfs, while only 20% of Mf3 and Mf4 were replaced 6 weeks after transplantation. However, after 1 year, all donor Mfs were replaced by recipient monocytes. These data elegantly showed that human intestinal Mf compartment is fully replenish through blood monocytes in a maximum of 1 year, and consists of transient immature Mfs (Mf1 and Mf2 subsets) that die or differentiate into long-lived mature Mfs (Mf3 and Mf4 subsets) (75) (Figure 6). This is in contradiction with mouse data, where it has been recently shown that there is a self-maintaining Mf population that persists throughout adulthood and is essential for intestinal homeostasis (232, 260).

To summarize, during homeostasis, circulating classical monocytes constantly replenish the intestinal Mf population, first constituting functionally plastic immature Mfs, which is a very appropriate way to respond rapidly and aggressively to pathogens through phagocytosis and cytokine secretion. Then, under the influence of the intestinal microenvironment, maturation steps generate long-lived anergic resident mature Mfs, which maintain oral tolerance and tissue homeostasis to prevent chronic inflammatory responses (Figure 2). The exact mechanisms involved in the Mf maturation waterfall remain to be fully elucidated in order to understand alterations during intestinal diseases such as IBD.

### Conventional Dendritic Cells in Intestinal Mucosa During Homeostasis

Before venturing deeper into the specifics of human intestinal cDC subsets, it is worth taking a moment to say that definition of these subsets is difficult. Indeed, based on data from mouse models where the integrins CD103 and CD11b are widely used to identify four intestinal cDC subsets (45, 261), many human studies use CD103 along with SIRPα (also called CD172a), as the human equivalent of CD11b in mice, to likewise identify four intestinal cDC subsets (45, 261). It has been shown that CD103^+^SIRPα^−^ were usually related to cDC1, CD103^+^SIRPα^+^ and CD103^−^ SIRPα^+^ were related to cDC2 while CD103^−^SIRPα^−^ cDCs are poorly studied (45, 75, 134, 220, 224). However, these markers do not robustly define cDC subsets in humans (132) and recent analyzes use other markers such as CLECL9A/CAMD1/CD26 and CD1c to characterize them (118, 135) (Figure 3C). Thus, the discussion below is divided to reflect these different ways to define human cDC subsets.

#### Frequency of Intestinal Mucosa cDC Subsets Defined Using CD103 and SIRPα

The frequency of CD103^+^SIRPα^+^ cells among cDCs predominated in small intestine but decreased in colon, while the contrary was true for CD103^−^SIRPα^+^ and CD103^−^SIRPα^−^ cells (75, 134, 220) (Figure 5C). Furthermore, the frequency of total CD103^+^ cDCs, as well as the frequency of CD103^+^ cells among each cDC subset, decreased in colon relative to ileum (132, 227). Thus, CD103-expressing cDCs decreased in colon relative to small intestine regardless of the subset analyzed. Importantly, the mechanisms regulating CD103 expression on cDCs are poorly known, suggesting that other markers could be more robust to defined human intestinal cDC subsets.

#### Phenotype and Frequency of Intestinal Mucosa cDC Subsets Using Markers Other Than CD103 and SIRPα

First, among MNPs, intestinal cDCs are characterized as CD14^−^CD11c^int−hi^ cells (Figures 3A, 4) (75, 132, 135, 224). Within this population, cDC1, cDC2, and DN DCs can be identified based on two major surface markers, CD1c and XCR1. Precisely, cDC2 are defined as CD1c^+^XCR1^−^, cDC1 as CD1c^−^XCR1^+^, and DN cDCs as CD1c^−^XCR1^−^ (Figures 3A, 4). In addition, these three cDC subsets expressed other specific markers as shown in Figure 1 (132, 134, 135, 224). DN cDCs seemed more heterogeneous with at least three other subsets based on SIRPα and CD26 expression (118, 134, 135) (Figure 3C). Regarding their abundance in control intestinal mucosa, cDCs represent 0.3–0.6% of colonic LP cells, which corresponds to a median of 114 cDCs per mg of tissue (229, 262). More precisely, our group showed that cDC1, cDC2, and DN cDCs represent around 0.05, 0.2, and 0.2%, respectively, of LP cells in both ileum and colon (132) (Figure 5A). However, among MNPs, the total cDC population seemed to decrease from ileum to colon (226) (Figure 5B). Finally, among cDCs, cDC1, cDC2, and DN cDCs represented around 10–15, 40–50, and 35–50%, respectively, both in ileum and colon (132) (Figure 5D).

To note, as described above in the blood, intestinal pDCs are present among the CD14^−^CD11c^−^ population and can be identified as CD123^+^CD45RA^+^ cells (135) (Figure 4). However, even if pDC frequency seems to increase by 10-fold from jejunum to colon, i.e., from 0.001 to 0.01% of CD45^+^ cells (135), human intestinal pDCs represent a very low amount of cells (135, 263), and will not be discussed further in this review.

#### Function of “Total” Intestinal Mucosa cDCs

In control intestinal mucosa, the frequency of cytokine-producing cDCs was absent/low for IL-6, IL-12, IL-22, and IL-23 and intermediate for IL-10, TGF-β, TNF, and IL-1β (220, 264). In addition, intestinal cDCs secreted almost no cytokines without stimulation and did not respond to TLR ligands *ex vivo* (75, 226). Supporting this, cDCs seemed immature as judged by their low expression of CD80, CD83, CD86, and TLRs (220, 226, 229, 264, 265). Mann et al. showed that functional differences could exist between colonic and ileal cDCs, such as induction the gut-homing receptor CCR9 (220), which makes the comparison of studies even more complex.

With respect to T cell induction, Ogino et al. showed that colonic cDCs induced naïve CD4^+^ T cell polarization into Th1 but not Th17 cells (226) (Table 1). In contrast, Mann et al. showed that colonic cDCs induced high production of several cytokines such as IL-10, TGF-β, IL-17, IFN-γ, and IL-22 by naïve CD4^+^ T cells (265), at least by dividing CFSE^lo^ T cells (Table 1). To note, Fenton et al. recently showed that intestinal cDCs that highly express integrin αVβ8, such as cDC2, but not cDC1, might induce higher Treg polarization through TGF-β secretion (266). Nevertheless, given that there are few naïve CD4^+^ T cells in human intestinal mucosa (257–259), it is more relevant to study naïve CD4^+^ T cell polarization with cDCs from mLNs, as discussed below.

**Table 1:** Th polarization by human intestinal cDCs.

**Type of tissue**	**Population of cDC**	**Th polarization**		**References**
		**Polarization of**	**No effect on**	
Colon	Total	Th1	Th17	(226)
Colon	Total	Th1 Th17 Th22 Treg (dividing CFSE low T cells)		(265)
Colon	CD103^+^	Treg	Th1 Th17	(227)
Jejunum	CD103^+^SIRPα^−^	Th17	Th1 Treg	(134)
	CD103^+^SIRPα^+^	Th17 Treg	Th1	
	CD103^−^SIRPα^+^	Th1	Th17 Treg	

#### Function of Intestinal Mucosa cDC Subsets Defined Using CD103 and SIRPα

Watchmaker et al. showed that CD103^+^SIRPα^−^, CD103^+^SIRPα^+^, and CD103^−^SIRPα^+^ in jejunum expressed a low level of CD80, an intermediate level of CD83 and CD86, while a high level of CD40 (134). In addition, they expressed CCR7, suggesting that these three cDC subsets may migrate to mLNs (134, 220). Furthermore, these cDC subsets induced the mucosa-associated integrin α4β7 and the gut-homing receptor CCR9 on naïve CD4^+^ T cells (134), suggesting that they support T cell homing to intestinal tissue. Matsuno et al. showed that colonic CD103^+^ cDCs induced Tregs, but neither Th1 nor Th17 (227) (Table 1). In contrast, Watchmaker et al. showed that both CD103^+^SIRPα^+^ cDCs and CD103^+^SIRPα^−^ cDCs induce Th17, while CD103^+^SIRPα^+^ cDCs induce Tregs and CD103^−^SIRPα^+^ cDCs induce Th1, at least with cDCs from jejunum of three obese subjects (134) (Table 1).

To note, Richter et al. recently described a monocyte-derived cell population in the cDC compartment, identified as HLA-DR^+^CD14^−/lo^CD11c^+^SIRPα^+^, which are mainly cDC2 (224). Indeed, some SIRPα^+^ cDCs expressing calprotectin and low level of CD14 were enriched in monocyte gene signatures, were morphologically similar to monocytes and did not express FLT3 receptor (224). Moreover, they exhibited a higher capacity for antigen processing, yet an inferior potential for migration and priming of naïve T cells compared to SIRPα^+^FLT3^+^calprotectin^−^ cDCs (224). These data suggest that CD14^lo^CD11c^+^SIRPα^+^FLT3^−^calprotectin^+^ cDCs, although mimicking a typical cDC phenotype, are more related to the monocyte lineage than to *bona fide* cDCs, highlighting once again the diversity and complexity of human intestinal MNP subsets. Finally, using pancreatico-duodenal transplantation patients, Richter et al. showed all intestinal cDCs were replaced by recipient cells 6 weeks after transplantation, suggesting that cDCs lack self-renewal capacity and long-life phenotype in the human intestine (224).

To summarize, the different gating strategies used and the low number of cDCs complicates getting a clear picture of subset-specific function. However, it has been shown that human intestinal cDCs are replenished by blood-derived cells in a few weeks, are poor cytokine-producing cells and play a role of sampling antigens to activate T cell proliferation in mLNs, which is consistent with the well-characterized overall function of cDCs (Figure 2). Nevertheless, additional studies of human intestinal cDCs are warranted to understand which and how cDC subsets maintain tissue homeostasis and initiating effective immunity without driving disease pathogenesis.

### Macrophages in Intestinal Mucosa During IBD

#### Phenotype and Frequency of Intestinal Mucosa Macrophages

It is now clear that the frequency and number of Mfs among intestinal LP cells is increased in IBD patients compared to controls, especially in active lesion areas (132, 221, 223, 225, 226, 228, 246, 263, 267–272) (Figure 5E). More specifically, the augmentation of intestinal Mfs is due to an increase in the frequency of immature Mfs among LP cells, despite that somewhat different surface markers and combinations thereof have been used to characterized them (i.e., HLA-DR or CD11c levels; scRNA-seq) (132, 221, 223, 225, 226, 228, 256, 263, 270, 272–275) (Figure 5F). This accumulation seems to be due to the inflammatory intestinal microenvironment of IBD patients, which boosts the recruitment of classical monocytes through mechanisms involving CCL2, IL-8, and TGF-β signaling (223, 276). These newly recruited monocytes are maintained in the immature pro-inflammatory state, which in turn amplify intestinal chronic inflammation (11, 277). Chapuy et al. showed that the frequency of immature Mfs, but not mature Mfs, is positively correlated with endoscopic score of disease severity in Crohn's patients (228). To note, age, gender, age at diagnosis, disease location and disease behavior, as well treatment history, did not influence the increased frequency of immature Mfs in Crohn's patients (228).

Some studies have addressed the interesting question of the effect of immunotherapy in IBD patients on the intestinal Mf compartment. For example, the frequency of immature Mfs decreased slightly in IBD patients after 5 weeks of anti-TNF adalimumab treatment (278). Specifically, this effect appears to be limited to patients in remission, at least after 14 weeks of infliximab therapy (279). Moreover, Vos et al. showed that the frequency of mature Mfs increased in IBD patients after 4 weeks of anti-TNF infliximab treatment (280). On the contrary, the two aforementioned studies did not find a change in the frequency of mature Mfs (278, 279). In addition, the frequency of immature Mfs decreased and the frequency of mature Mfs increased in IBD patients after 14 weeks of anti-α4β7 vedolizumab treatment, specifically in patients in remission (279). Even if these three studies have not used the same markers to define Mf subsets, their data go in the same direction. That is, reduced immature Mf and/or increased mature Mf frequencies accompany disease quiescence after immunotherapy. This suggests that the restoration of homeostatic Mf composition is resulting from and/or is necessary during the remission of IBD patients after immunotherapy.

A fundamental question that remains is which components of the intestinal microenvironment drive Mf maturation during homeostasis, and how this process is affected during IBD. One candidate could be GM-CSF. Indeed, it has been shown that blood monocytes differentiate to immature Mf-like cells through GM-CSF *ex vivo* (281, 282), a factor that increases in Crohn's disease and UC, particularly in active lesion areas (283). Moreover, GM-CSF can act in concert with IFN-γ and TNF to reprogram blood monocytes into Mfs with an inflammatory profile (284). However, GM-CSF has also been shown to induce blood monocytes with a tissue repair and anti-inflammatory profile, which can dampen intestinal inflammation in mouse models (285). This mechanism could contribute to the benefit of GM-CSF therapy observed in some Crohn's patients (286). Therefore, either the absence or chronic production of GM-CSF can result in high susceptibility to intestinal pathology, demonstrating the importance of its balanced production to maintain homeostasis. However, the regulation of Mf function goes far beyond a single factor. Indeed, it is difficult to imagine the scenario when numerous immunomodulating factors such as IFN-γ, TNF, IL-1β, IL-6, IL-36, TGF-β, and IL-10 are present in various combinations and amounts, as is the case *in vivo* (11). Clearly, this area of research warrants further investigation.

#### Function of Intestinal Mucosa Macrophages

In addition to their accumulation, intestinal Mfs produced more pro-inflammatory cytokines, such as TNF, IL-23, IL-1β and IL-6, in basal conditions as well as after TLR stimulation, in UC patients and even more in Crohn's patients compared to controls (226, 228, 246, 256, 271, 287). These pro-inflammatory cytokines can promote and/or perpetuate a pathologic environment (11). For example, Takayama et al. showed that Mfs from Crohn's patients induced IFN-γ secretion by NK cells via IL-23 release and cell-cell contact (288). Moreover, factors in LP-conditioned medium from Crohn's patients, including IFN-γ, induced inflammatory monocyte differentiation and IL-23 secretion by these cells, leading to a vicious circle perpetuating inflammation (246). Importantly, Mfs from Crohn's patients also expressed higher levels of both IL-10 and latent TGF-β, which have anti-inflammatory effects (226, 256). Nevertheless, Kelly et al. recently showed that integrin αvβ8 expression, which regulates immune tolerance via TGF-β activation as discussed above, was highly reduced on Mfs from IBD patients (244). This suggests that, even if Mfs from Crohn's patients expressed more latent TGF-β, less TGF-β was in the active form (244).

Additional mechanisms that amply inflammation may also contribute to IBD pathogenesis. For example, the frequency and the number of TREM-1^+^ Mfs, which are mainly immature Mfs, are increased in IBD patients, especially in active lesion areas (254, 255). As described above, TREM-1 is an inflammation-amplifying receptor expressed on myeloid cells; it is involved in immune responses triggered by bacteria (249, 250), yet its role in IBD is poorly understood. Our group showed that an anti-TREM-1 antagonist antibody dampened secretion of several pro-inflammatory cytokines and chemokines by LP cells from highly inflamed intestinal mucosa of IBD patients (255), supporting a potential role of TREM-1 in perpetuating intestinal inflammation in IBD patients.

Other disease-associated changes in Mf function may promote IBD. For example, it has been suggested that intestinal Mfs have bacterial clearance impairment in IBD patients (289), and in patients manifesting a Crohn's disease phenotype, i.e., Niemann–Pick disease type C1, mainly through dysfunctional autophagy (290). Moreover, they appear to contribute to intestinal barrier dysfunction. Indeed, Mfs from inflamed Crohn's disease tissue induced less IL-22 secretion by innate lymphoid cells than those from quiescent area (248), and blood monocytes reduced epithelial barrier efficiency *in vitro* by altering the structure and function of tight junctions (271). Finally, intestinal Mfs from IBD patients had increased ROS production (291), which can also contribute to epithelial injury (292).

Another role of intestinal Mfs is their involvement in tissue repair and fibrosis (99, 293). To note, at least 10% of Crohn's patients have an intestinal fibrostenosis phenotype at the time of diagnosis (294). In addition, fibrotic complications, such as strictures, occur in ~20–30% of Crohn's patients 10 years after diagnosis (294). In UC patients, the degree of fibrosis is proportional to the degree of inflammation, even if the fibrosis-associated strictures are less prevalent than in Crohn's patients (294). Despite the problem of fibrosis in IBD (295, 296), the mechanism by which Mfs contribute to fibrosis-associated pathology is poorly understood. Several Crohn's-associated susceptibility loci, including some related to MNP-associated inflammation such as NOD2, ATG16L1, IL-12B, IL-23R, and CX3CR1, are predictors of fibrostenosis (294). In addition, Scheibe et al. showed an increase number of intestinal IL-36α^+^ Mfs in IBD patients, which correlated with the degree of inflammation (297) and the accumulation of αSMA^+^ myofibroblasts (298). More precisely, the number of intestinal IL-36α^+^ Mfs increase in the colon of Crohn's patients with stenosis (298). Functionally, IL-36 acted directly on human mesenchymal cells to elicit a profibrotic transcriptional program (298), suggesting that the increase of IL-36α^+^ Mfs could induce intestinal fibrosis during chronic inflammation in IBD patients (298–300). Corroborating this, Martin et al. recently showed *in situ* that immature Mfs were always in the close vicinity of activated fibroblasts in intestinal mucosa of Crohn's patients (263). In particular, immature Mfs, as well as cDC2, induced intestinal inflammation through fibroblast activation via oncostatin M/OSMR signaling, which increased in IBD patients and predicted anti-TNF therapy response (275, 301). However, even if OSM induced IL-11 expression by activated fibroblast (275), which is known to be a major fibrotic component (302), whether OSM promotes intestinal fibrosis in IBD patients remains to be determined.

Regarding T cell activation, intestinal Mfs from Crohn's patients induced naïve CD4^+^ T cell proliferation as well as integrin β7 and CCR9 expression in the same range as those from controls (287). However, Barman et al. showed that mature Mfs from UC patients were unable to suppress effector T cell proliferation compared to those from controls (256) (Figure 2). In addition, intestinal Mfs from Crohn's patients induced more Th1 and Th17 polarization from naïve CD4^+^ T cells (226, 287) (Figure 2). This seems to be due to immature Mf accumulation within the total Mf population in Crohn's patients. Indeed, Chapuy et al. have recently shown that immature Mfs from IBD patients, but seemingly not mature Mfs, induce Th17 cells, as well pathologic IFN-y^+^IL-17^+^ T cells (303), from autologous colonic CD4^+^ T cells mainly through their production of IL-1β (228, 272). Corroborating this, Martin et al. have shown that, while initial steps of lymphocyte aggregate formation depend on DCs, immature Mfs likely participate in T cell activation *in situ* (263).

In summary, data support that there is a large influx of inflammatory immature Mfs that drive inflammation and tissue damage in IBD (Figure 2). Moreover, although mature Mfs seem to maintain their anti-inflammatory and tissue repair functions in IBD, their relative abundance is reduced during inflammation as immature Mfs dominate. However, it is still unclear if the disruption of blood monocyte differentiation into mature Mfs reflects a loss of intrinsic maturation cues that normally program recruited monocytes toward cells with tolerogenic properties or if the chronic inflammatory microenvironment generates new factors that actively overhaul this homeostatic process. Thus, development of new therapies to restore the Mf maturation process and/or neutralize factors that drive monocyte recruitment may be beneficial for treating IBD.

### Conventional Dendritic Cells in Intestinal Mucosa During IBD

#### Phenotype and Frequency of Intestinal Mucosa cDC Subsets

Several studies found no difference in the number, frequency or maturation state (with respect to CD80, CD83, and CD86 levels) of total intestinal cDCs, as well cDC1 and cDC2 subsets, in active lesion areas of IBD patients compared to quiescent lesion areas as well compared to controls (132, 229, 263, 269, 278) (Figure 5G). These data contrast a single study showing increased cDC2 among LP cells of IBD patients (262). To note, using scRNA-seq, Martin et al. have recently defined four cDC subsets in ileum from Crohn's patients, namely cDC1, cDC2, monocyte-derived DC-like cells and activated cDCs (263). Activated cDCs expressed CCR7 and PD-L1, as well as the lymphocyte-attracting chemokines CCL17, CCL22, and CCL19, and was the only cDC subset increased in inflamed lesions compared to uninflamed lesions (263).

Given the caveat that CD103 expression does not robustly define functionally distinct subsets of human intestinal cDCs, as discussed above, it has been shown that cDCs expressing CD103 are decreased among LP cells, as well as among MNPs, in both Crohn's and UC patients (132, 227, 278). In addition, the frequency of CD103^+^ cells among cDC1, cDC2 and DN cDCs subsets was lower in active IBD intestinal tissue compared to quiescent tissue, and even more so compared to controls (132). However, mechanisms underlying reduced human intestinal CD103^+^ cDC frequency in IBD patients are not known. It could be due to inflammation-induced cell death, downregulation of CD103 expression and/or emigration of CD103^+^ cDCs from intestinal LP. Thus, further investigation to understand CD103 regulation in human intestinal cDCs, its role on cDC function and its possible impairment during IBD are needed.

#### Function of Intestinal Mucosa cDC Subsets

Several studies showed that intestinal cDCs have a higher inflammatory state in UC patients, and even more so in Crohn's patients, compared to controls. This is supported by the increased frequency of CD40^+^, TLR2^+^, TLR4^+^, IL-12^+^ and IL-6^+^ cDCs, but not IL-10^+^ cDCs in patients' tissues (262, 264, 265, 304). Consistent with this, LPS-stimulated intestinal cDCs from Crohn's and UC patients secreted more TNF and IL-8 compared to those from controls (262). In addition, the frequency of IL-6^+^ cDCs and TLR4^+^ cDCs were associated with the Crohn's Disease Activity Index (304). To note, even if the frequency of CD103^+^ cDCs decreased in UC compared to controls, they were more inflammatory and induced less Tregs but more Th1, Th2, and Th17 polarization of naïve CD4^+^ T cells (227).

As for intestinal Mfs, the factors that trigger the inflammatory state of cDCs in the intestine of IBD patients are not fully understood. Wu et al. showed that TNF and IFN-γ reduced miR-10a expression in DCs from IBD patients, resulting in enhanced IL-12/23p40 and NOD2 expression as well as Th1 and Th17 polarization (305). The microbiome composition may also influence DC function, as suggested by Ng et al. (304). Indeed, in this study, the frequency of IL-12p40^+^ DCs positively correlated with Bacteroides and the frequency of IL-6^+^ DCs negatively correlated with *F. prausnitzii*, which are considered detrimental and beneficial, respectively, during IBD (304). Thus, although a cause/effect relationship between dysbiosis and altered DC function is not established, these data raise the possibility that, during IBD, intestinal dysbiosis drives higher production of pro-inflammatory cytokines by intestinal cDCs which, in turn, overcomes their regulatory properties and tips the balance toward inflammation (304).

Regarding T cell induction, intestinal cDCs from UC patients induced less T cell proliferation and the same amount of integrin β7 but more CCR9 on naïve CD4^+^ T cells compared to those from controls (265). Moreover, the dividing CFSE^lo^ CD4^+^ T cells produced less IFN-y and IL-22, similar IL-10, TGF-β and IL-17, but more IL-4 when co-cultured with cDCs from UC patients compared to cDCs from controls (265). To note, some of these alterations have been restored to control levels in the presence of *Lactobacillus casei* Shirota, a bacterium found in the commensal microbiota and used as a probiotic (265). This again suggests a role of microbiota in intestinal cDC regulation. Interestingly, Martin et al. showed that activated cDCs, which expressed lymphocyte-attracting chemokines, formed dense aggregates with lymphocytes (263). Moreover, both activated cDCs and Ki-67^+^ cycling lymphocytes were enriched in Crohn's patients with a “high inflammatory signature,” suggesting a role for activated DCs in the recruitment, local activation, expansion, and spatial organization of adaptive immune responses in inflamed lesions of Crohn's patients (263). Finally, Fenton et al. showed that the frequency of αVβ8^+^ cDC2, which are thought to induce Tregs, doubled in Crohn's patients compared to controls (266). However, intestinal Treg frequency among CD4^+^ T cells decreased in Crohn's patients (306). Nevertheless, given recent evidence that enhancing the ability of intestinal T cells to sense active TGF-β is effective in inducing remission in some Crohn's patients (307), boosting the αVβ8–TGF-β pathway may be an attractive complementary therapeutic approach to weaken inflammatory T cell responses (266).

#### Retinoic Acid Influence on Human Intestinal Mucosa MNP Subsets

A specific factor derived from dietary vitamin A1, retinoic acid (RA), has been shown to be a regulator of intestinal MNP functions (308). More precisely, RA release by cDCs has been related to Treg polarization, especially in mouse models (309), but it can be pro- or anti-inflammatory depending on the local microenvironment (310). In humans, intestinal Mfs and cDC subsets had RALDH activity and expressed ALDH1A1, ALDH1A2, and/or RDH10 suggesting that they possess the complete enzymatic machinery to generate RA from vitamin A1 (132, 134, 222, 287). Moreover, they induced the gut-homing α4β7 and CCR9 on naïve CD4^+^ T cells in a RA-dependent manner (222, 287). To note, there was no difference between ileal or colonic cDCs, or between cDC subsets, concerning RALDH activity and induction of α4β7 and CCR9 on naïve CD4^+^ T cells (132, 134, 222). This contrasts data from mice (309, 311).

Regarding RALDH activity by intestinal MNPs during IBD, there are conflicting data (132, 222, 287). For example, Sanders et al. showed that intestinal Mfs, and both CD103^+^ and CD103^−^ cDC subsets, from Crohn's patients had higher RALDH activity compared to controls (222). In contrast, Magnusson et al. showed that RALDH activity in intestinal Mfs, cDC1, and cDC2 subsets decreased in UC patients compared to controls, and the same trend was observed in Crohn's patients although not reaching statistical significant (132). In addition, there are also discrepancies regarding ALDH1A1, ALDH1A2, and/or RDH10 gene expression between these three studies. For example, Kamada et al. observed the same level of RALDH2 expression but a decrease of RDH10 expression by intestinal Mfs from Crohn's patients compared to those from controls (287). Furthermore, they showed that intestinal Mfs from Crohn's patients induced more Th17 polarization from naïve CD4^+^ T cells compared to those from controls and that RA can act as a suppressor of this Th17 polarization (287). Therefore, they suggested that an RA-dependent Th17 polarization suppressive pathway was impaired in intestinal Mfs from Crohn's disease patients (287). However, others found no difference, or even an increase, in ALDH1A1, ALDH1A2, and/or RDH10 gene expression in MNP subsets from Crohn's patients compared to controls (132, 222). Nevertheless, none of these three studies measured RA itself nor assessed other factors that regulate RA availability. Additionally, given that intestinal T cells are almost entirely memory T cells (257, 258), the biological significance of the ability of RA-producing intestinal MNPs to regulate naïve CD4^+^ T cells as shown *ex vivo* is not clear. To conclude, it is more relevant to analyze this property in mLN MNP subsets, especially cDCs, as described below.

To summarize, in most tissues, exposure to microbial components is sufficient to induce inflammatory cDCs, while in the intestine additional signals are required due to the necessity to maintain homeostasis in this microbe-rich environment. Thus, inflammation-dampening mechanisms in cDCs must be overcome to enable them to drive inflammation in the intestine. Even if the additional signals are not defined, they likely increase during IBD and lead to pro-inflammatory cytokine production by cDCs (Figure 2). However, it is unclear if these inflammatory/activated cDCs arise from modulation of local cDC populations, recruitment of blood cDCs, differentiation of monocyte-derived cDCs, or a combination of these scenarios. Moreover, the non-redundant roles of the cDC1 and cDC2 subsets during IBD, such as mLN migration capacity and Th polarization, are poorly understood. Therefore, better understanding of intestinal cDC dysregulation is required to target these cells as a means to treat IBD.

## Human Mesenteric Lymph Node MNP Populations During Homeostasis and IBD

### Macrophages in Mesenteric Lymph Nodes

Since Mfs are primarily involved in non-lymphoid tissues, there is little data available on Mfs from mLNs, especially in humans. Granot et al. showed that the Mf population represents 0.8% of CD45^+^ cells in mLNs from control individuals (135). In IBD patients, the frequency of Mfs in mLNs increases and they produced more IL-1β and TNF compared to those from controls (268, 312, 313). Chapuy et al. recently showed that mLN Mfs had high frequency of cells positive for inflammatory cytokines compared to mLN SIRPα^+^ DCs, which contain a mix of cDC2 and pDCs (313). However, these mLN Mfs poorly activated naïve T cells and did not contribute to Th17 plasticity toward Th1 and Th1/Th17 profiles compared to mLN SIRPα^+^ DCs (313). Finally, mLN Mfs highly expressed genes involved in phagocytosis and in leucocyte chemotaxis (313). These data corroborated with those from mice, where it has been shown that mLN Mfs poorly activate naïve T cells and were involved in clearing apoptotic cells and promoting B cell activation (314, 315). To note, using CyTOF, Chapuy et al. described 7 mLN Mf subsets in IBD patients (313), but their location, function and disease involvement remain to be deciphered.

### Conventional Dendritic Cells in Mesenteric Lymph Nodes During Homeostasis

In mLNs, as in the other draining LNs, there are two cDC subpopulations: resident immature cDCs derived directly from the blood (called resident cDCs hereafter) and migratory mature cDCs derived from intestinal mucosa (called migratory cDCs hereafter) (135, 316, 317). Both populations are CD45^+^Lin^−(CD3/CD19/CD56)^HLA-DR^+^CD11c^+^CD14^−^ but differ in the level of HLA-DR and CD11c expression. That is, resident cDCs are HLA-DR^int^CD11c^hi^ while migratory cDCs are HLA-DR^hi^CD11c^int^ (Figure 3D). In contrast to lymphoid organs lacking afferent lymphatic vessels, such as the spleen and tonsils, where resident cDCs represent the vast majority (~98%) of the cDC fraction, resident cDCs represent 50 to 90% of total cDCs in draining LNs (135, 143).

#### Phenotype and Frequency of mLN cDCs

Both resident and migratory cDCs have been identified in mLNs of control individuals, but the percentage of each differs between studies (132, 135, 318) (Table 2). Indeed, Magnusson et al. observed a 7:1 migratory: resident cDC ratio in ileal mLNs (132), while Sakuraba et al. reported a 1:1 ratio in colonic mLNs (318) (Table 2). Recently Granot et al. found a 1:5 ratio in mLNs from unspecified intestinal regions (135) (Table 2). These discrepancies could be due to several reasons such as flow cytometry gating strategies or the “type” of control patients (Table 2). Thus, so far, available data do not allow a clear picture regarding the migratory:resident cDC ratio in mLNs during homeostasis. Regarding cDC subsets, Granot et al. found that cDC1 and cDC2 represent 0.05 and 0.1% of CD45^+^ cells in mLNs, respectively (135). More precisely, they observed a 3:1 cDC2/cDC1 ratio among resident cDCs while a 20:1 cDC2/cDC1 ratio among migratory cDCs (135) (Table 2). Magnusson et al. also showed a higher cDC2/cDC1 ratio among migratory cDCs compared to resident cDCs (132), even if it was not in the same range as that of Granot et al. (Table 2). Nevertheless, it seems that cDC2 represent the major subset of migratory cDCs in human mLNs (Figure 3D). However, migratory cDC1 may still go unnoticed by flow cytometry analysis if they downregulated their markers upon migration or/and die shortly after reaching mLNs (316). Thus, it remains to be determined if there is an imbalance between cDC2 and cDC1 migration *per se* to the mLNs, or if the proportional differences in mLNs simply reflects the relative frequencies that already exist in the intestinal mucosa.

**Table 2:** Characteristics of human mLN cDCs during homeostasis and IBD.

**Reference**	**(****318****)**		**(****132****)**		**(135)**
**“Type” of patients**	**Colorectal cancer**	**CD and UC**	**Bladder reconstruction**	**CD and UC**	**Deceased organ donors**
Number of subjects	3	3 and 3	4	10 and 5	30
Intestinal region	colon	Ileum and colon	Ileum	Ileum and colon	Unspecified
Migratory: resident cDC ratio	1:1	1:1	7:1	1:1	1:5
cDC2/cDC1 ratio among resident cDCs	n.a		2:1	4:1	3:1
cDC2/cDC1 ratio among migratory cDCs	n.a		4:1	6:1	20:1

#### Function of mLN cDCs

First, contrary to dogma, Sakuraba et al. showed that resident and migratory cDCs induce the same release of IFN-γ, IL-4, and IL-10 by naïve CD4^+^ T cells (318). Regarding functional assays on cDC subsets, Jaensson et al. showed that CD103^+^ cDCs, which represented around 30% of cDCs in mLNs, were more mature than CD103^−^ cDCs, as judged by expression of CD40 and CD83 (311). In addition, CD103^+^ cDCs seem more inclined to immunosuppressive effects and induce more naïve CD4^+^ T cell polarization toward Tregs (319, 320). However, concerning RA activity, which is considered to be involved in Treg polarization in intestinal mucosa as discussed above, Sato et al. showed that neither human mLN CD103^+^ cDCs nor CD103^−^ cDCs have *ex vivo* ALDH activity at basal state (321). This contrasts data from mice (309, 322). In addition, after *ex vivo* stimulation with GM-CSF, RA, and Vitamin D3, CD103^−^ cDCs had more ALDH activity than CD103^+^ cDCs (321). Thus, as it has been shown in mouse skin-draining LN cDCs (323), CD103 expression does not constitute a marker for RA-producing human mLN cDCs. Thus, even if mLN CD103^+^ cDCs seem more tolerogenic than CD103^−^ cDCs, the mechanisms for this are unknown. Moreover, analyzes need to be performed on cDC subsets delineated with more robust phenotypic markers.

### Conventional Dendritic Cells in Mesenteric Lymph Nodes During IBD

#### Phenotype and Frequency of mLN cDCs

Concerning the migratory: resident cDC ratio in mLNs, Sakuraba et al. reported no difference between controls and IBD patients, which was 1:1 in both groups (318) (Table 2). In contrast, Magnusson et al. showed that this ratio decreased from 7:1 to 1:1 (132) (Table 2). Regarding cDC subsets, it has been shown that the cDC2/cDC1 ratio, among both resident and migratory cDCs, tends to increase in mLNs of IBD patients (132) (Table 2). Additional work is needed to clarify the migratory: resident cDC ratio and the cDC2/cDC1 ratio between controls and IBD patients, as well as to understand the significance of any revealed differences and the mechanisms driving ratio alterations in health vs. IBD.

#### Function of mLN cDCs

Sakuraba et al. showed that total mLN cDCs from IBD patients release little IL-12, IL-23, and IL-10 with or without LPS treatment *ex vivo* (318). However, in response to *Enterococcus faecalis* extract, the release of these cytokines increased in both Crohn's disease and UC patients (318). MLN cDCs from Crohn's patients induced more Th1 but similar Th2, Th17, and Treg polarization from naïve CD4^+^ T cells compared to those from controls or UC patients (318) (Figure 2, dotted square lower right). Regarding mLN CD103^+^ and CD103^−^ cDCs, there was no difference in CCR9 and α4β7 induction on CD8^+^ T cells (311) or in ALDH activity (321) between cells from Crohn's patients compared to those from controls. In summary, much work remains to decipher the function of the cDC network, including the role of *bona fide* cDC1 and cDC2 subsets identified using robust markers, in mLNs during homeostasis and IBD pathogenesis.

## Concluding Remarks and Future Perspectives

### What Is the Current Status of Human Intestinal MNP Studies?

Despite the recent advances that have furthered our understanding of human intestinal MNPs, in both homeostasis and IBD, many important questions remain. In particular, intestinal MNP subsets seem to have both overlapping and distinct functional abilities, and unraveling this complexity is definitely a challenge. This is exemplified by the absence of a clear picture of T cell skewing capacities of MNP subsets despite experimental efforts. Part of the difficulty in understanding MNP function may be the spectrum of MNP subsets, which can complicate comparison of data from different research groups and lead to discrepancies (45, 261, 324, 325). Moreover, intestinal MNP regulation is complex and influenced by other immune cells, epithelial cells, and stromal cells (263, 274, 275, 326), as well as microbiota and metabolic components (17, 327, 328). Nevertheless, it is clear that the increase of immature Mfs and activated cDCs play a major role in IBD pathogenesis given their production of inflammatory cytokines/chemokines and their activation of stromal cells promoting their own recruitment and perpetuating an inflammatory cycle that leads to intestinal damage.

### Can We Improve Conventional Strategies to Treat IBD?

Therapeutics for IBD that suppress intestinal inflammation by cytokine blockade have been used for some time (329), and ongoing testing of new drugs that target cytokines supports that this treatment avenue will remain viable (11, 330, 331). However, important questions remain. For example, do robust biomarkers exist to predict efficacy prior to initiating a treatment? What are the prospects of developing predictive biomarkers for existing therapies where they are lacking, and for new therapies as they enter the clinic? Will we be able to screen patients and stratify them for appropriate therapies?

The most significant breakthrough thus far in treating IBD, and is perhaps the classic example of suppressing a pro-inflammatory cytokine, is anti-TNF treatment. However, neutralizing TNF by no means helps all patients, as 30% of patients do not respond 1 year after treatment (332). Moreover, most of strategies targeting single effector cytokines in IBD have been disappointing in clinical trials (333), reinforcing the complexity and heterogeneity of IBD. Indeed, as many types of intestinal cells produce a wide range of effector cytokines, it is not surprising that it may be necessary to simultaneously target multiple cytokines to effectively suppress intestinal pathology. This is supported by positive effects of concomitant blockade of IL-12 and IL-23 using a monoclonal antibody against the IL-12p40 subunit (11, 330, 331, 333–339).

In addition to strategies that neutralize pro-inflammatory cytokines, an alternate approach has been to promote anti-inflammatory responses through application of cytokines such as IL-2, IL-10, IL-22, or TGF- β1 (11, 331, 336). Although these have been promising to some extent in animal models, they have not undergone rigorous clinical trials. For example, directly targeting TGF-β1 could be difficult given its multiple functions that distinctly influence the disease; nevertheless, recent clinical studies showed the efficacy of TGF-β signal restoration in IBD (307, 340, 341). Moreover, promoting intestinal repair through IL-22Fc could be another way to reduce burden in IBD patients (342).

One more pillar in IBD therapy is blocking immune cell trafficking molecules, such as α4β7 and MadCAM-1, as a general way to prevent influx of potentially pathogenic cells into the intestine. This approach has shown promise in experimental models but has had limited success in treating IBD patients (279, 331, 343). Nonetheless, etrolizumab anti-β7, which blocks both α4β7/MAdCAM-1 and αEβ7/E-cadherin interaction, is currently under evaluation in a phase III trial (344, 345). Another interesting way could be to do the opposite. Indeed, activating the integrin Mac-1 (CD11b/CD18) with small-molecule agonists inhibited immune cell recruitment by increasing their adhesion to the inflamed endothelium, at least in mice (346, 347). Therefore, development of therapies that target immune cell trafficking in intestine of IBD patients is attractive.

### Can Specific MNP Populations Be Targeted to Treat IBD?

#### Monocytes and Macrophages

Significant data support that newly recruited monocytes in inflamed intestinal mucosa are central to driving the immunopathogenesis of IBD. Thus, developing treatments that dampen the number and/or pathologic function of these cells is an exciting avenue to pursue. Therefore, targeting the CCR2-CCL2/CCL7 axis could be one possibility to block monocyte recruitment to the intestine (263, 348). However, this could also inhibit their recruitment to other tissues, thus inducing side effects. Additionally, an overall lack of intestinal Mfs would likely increase susceptibility to infections as well as suppress benefits of their tissue repair activity (349, 350). The potential risks associated with this type of immunotherapy necessitate careful monitoring programs.

Other approaches are to decrease inflammatory programs in intestinal Mfs. One possibility among other examples reviewed previously (11, 325) could be the inflammation-amplifying TREM-1 receptor expressed by Mfs (249–251). Indeed, it has been shown that TREM-1 plays a role in initial signaling toward an inflammatory state of newly recruited monocytes in a mouse model of small intestine inflammation (351), and inhibition of TREM-1/CLEC5A pathways reduced intestinal inflammation during colitis (254, 352). In humans, TREM-1^+^ Mfs are increased in IBD patients and its engagement enhanced pro-inflammatory cytokine secretion while a TREM-1 antagonist dampened it (254, 255). Finally, Verstockt et al. have shown that low TREM-1 expression in both whole blood and intestinal mucosa can predict anti-TNF therapy responders (353, 354). Thus, TREM-1 could be a potential biomarker for predicting the effect of anti-TNF therapy and, secondly, blocking TREM-1 could be an attractive target for IBD treatment.

Other ways to improve intestinal homeostasis could be to promote anti-inflammatory and pro-resolving functions in Mfs. This could be achieved, for example, by enhancing negative regulation of TLR signaling and silent clearance of apoptotic cells (33, 355–359), as well as by augmenting IL-10 receptor signaling (360–362). To note, thanks to their high phagocytic capacity, targeting intestinal Mfs could be facilitated by “delivery systems” such as nanomaterials and biomaterials (363), as has been shown in mouse model of organ transplantation (364). Finally, reprogramming of Mf using metabolites could become a promising approach to dampen intestinal inflammation. For instance, the short-chain fatty acid n-butyrate (365, 366) and itaconate (327, 367) induce anti-inflammatory program in Mfs, which could represent an opportunity to treat IBD (368).

#### Conventional Dendritic Cells

In view of the data from preclinical models of IBD supporting a role of cDCs in intestinal inflammation (11, 45), it is appealing to speculate that selectively targeting cDCs could be a treatment strategy. However, the main function of cDCs is in draining lymph nodes where they influence T cell skewing and imprint tissue homing properties (317). Therapeutic approaches targeting cDC function in IBD would optimally influence cDC function, preferably specifically in mLNs, which is challenging but potentially possible (369). However, intestinal cDCs rapidly respond to environmental cues, including dietary changes and pathogen exposure, suggesting their environmental responsiveness could be exploited to influence their function. They also produce and respond to key cytokines implicated in IBD pathogenesis, and other drug-sensitive pathways that can potentially be exploited to modulate cDC function for therapeutic approaches also exist (11). This could include prevention of cDC activation by blockade of activating and/or survival signals, interference with intracellular signaling pathways, neutralization of effector cytokines they produce, or perturbation of cDC trafficking to target organs, which in the case of IBD is the intestinal mucosa and mLNs. Important questions essentially unexplored are the effect of existing treatments on cDC number and cDC subset ratio, and if these are disease-relevant, as well as cDC function, which likely has implications on tipping the balance from health to disease.

## Conclusion

To conclude, advancing our understanding of the extended family of MNPs and dissecting their interactions with other cells comprising the networks that drive IBD is crucial to develop additional strategies to alleviate the chronic inflammation that underlies this debilitating disease. Indeed, recent publications using multi-dimensional analyses revealed cellular networks, including MNP subsets, involved in IBD pathogenesis, providing a platform for future therapeutic development. Finally, better characterization of pathophysiology in subgroups of IBD patients and developing combined immunotherapies for stratified, or possibly personalized, strategies is required to limit disease progression and develop new treatments for patients where current therapies are ineffective.

## Author Contributions

All authors listed have made a substantial, direct and intellectual contribution to the work, and approved it for publication.

### Conflict of Interest

The authors declare that the research was conducted in the absence of any commercial or financial relationships that could be construed as a potential conflict of interest.
